# Experiential Family Intervention for Children and Youth

**DOI:** 10.1192/j.eurpsy.2022.1921

**Published:** 2022-09-01

**Authors:** I. Guillemet, B. Jackson

**Affiliations:** 1 Private Practice, Psychiatry Child Adolescent, Paris, France; 2 Children Hospital, Psychiatry, Denver, United States of America

**Keywords:** Child Adolescent Family Therapy

## Abstract

**Introduction:**

Reviews suggest that family interventions including family therapy are effective for a range of disorders in youth. Family sculpting is used in different clinical settings to help young patients, their parents and siblings when words are not enough.

**Objectives:**

Participants will be able to understand the clinical relevance of family sculpting: shifting from discussions about family problems to physical representations of family dynamics and how to apply in their practice.

**Methods:**

There will be a brief overview of the general principle of family sculpting followed by clinical vignettes of patients combined with videos of the intervention. These examples will guide the discussion on how relevant in our clinical work this therapeutic practice may be. This variation on sculpting incorporates theater warmup exercises and therapists joining the family experience.

**Results:**

Family sculpting captures an immediate picture of the family dynamics that is a therapeutic turning point for families and gives voice to the children. The clinical cases and videos will guide clinicians on how to integrate into their own practice.

**Conclusions:**

This presentation will make possible integrating family sculpting into your own practice, providing an engaging alternative modality for complex cases.

**Disclosure:**

No significant relationships.

